# The 4 Youth By Youth mHealth Photo Verification App for HIV Self-testing in Nigeria: Qualitative Analysis of User Experiences

**DOI:** 10.2196/25824

**Published:** 2021-11-17

**Authors:** David Oladele, Juliet Iwelunmor, Titilola Gbajabiamila, Chisom Obiezu-Umeh, Jane Ogoamaka Okwuzu, Ucheoma Nwaozuru, Adesola Zaidat Musa, Ifeoma Idigbe, Kadija Tahlil, Weiming Tang, Donaldson F Conserve, Nora E Rosenberg, Agatha N David, Joseph Tucker, Oliver Ezechi

**Affiliations:** 1 Clinical Sciences Department Nigerian Institute of Medical Research Lagos Nigeria; 2 Department of Behavioral Science and Health Education Saint Louis University Saint Louis, MO United States; 3 Department of Epidemiology University of North Carolina at Chapel Hill Chapel Hill, NC United States; 4 Dermatology Hospital Southern Medical University Guangzhou China; 5 Department of Health Promotion, Education, and Behavior, Arnold School of Public Health University of South Carolina Columbia, SC United States; 6 Department of Medicine University of North Carolina at Chapel Hill Chapel Hill,, NC United States; 7 Faculty of Infectious and Tropical Diseases London School of Hygiene and Tropical Medicine London United Kingdom

**Keywords:** HIV self-testing, adolescents, young people, photo verification, mobile app, Nigeria

## Abstract

**Background:**

Despite the global expansion of HIV self-testing (HIVST), many research studies still rely on self-reported outcomes. New HIVST verification methods are needed, especially in resource-limited settings.

**Objective:**

This study aims to evaluate the user experience of a mobile health (mHealth) app to enhance HIVST result reporting and verification.

**Methods:**

Semistructured, in-depth interviews were used to evaluate the user experience of the 4 Youth By Youth mHealth photo verification app for HIVST. We used a think-aloud approach, and participants performed usability tasks and completed a qualitative exit interview. The app included HIV educational resources, step-by-step video instructions for performing HIVST, a 20-minute timer, a guide on interpreting results with linkages to care, an offline version, and a photo verification system. Demographic characteristics were reported by using descriptive statistics. Qualitative data were analyzed by using thematic analysis.

**Results:**

A total of 19 users—12 women and 7 men—with a mean age of 22 years, participated in the study. The users completed the usability tasks and successfully uploaded a photo of their test results by using the app without assistance. Four main themes were identified in the data. First, in terms of user-friendly design, the participants noted the user-friendly features of the offline version and the app’s low data use. However, some wanted the app to work in the background when using their mobile phone, and the font used should be more youth friendly. Second, in terms of ease of use, participants remarked that the app’s self-explanatory nature and instructions that guided them on how to use the app enhanced its use. Third, in terms of a user’s privacy, many participants reinforced the importance of privacy settings and tools that protect confidentiality among users. Finally, in terms of linkage to care, participants noted that the app’s *linkage to care* features were useful, particularly in relation to referrals to trained counselors upon the completion of the test. All the participants noted that the app provided a convenient and private means of verifying the HIV test results.

**Conclusions:**

Our findings demonstrated the importance of engaging end users in the development phase of health technology innovations that serve youth. Clinical trials are needed to determine the efficacy of using an mHealth app to verify HIVST results among young people.

## Introduction

Meeting the global 95-95-95 targets for HIV prevention among youth in sub-Saharan Africa requires innovation [1]. For example, in Nigeria, according to the 2019 National AIDS Indicator and Impact Survey report, there were 275,604 young people aged between 15 and 24 years living with HIV [2]. HIV self-testing (HIVST) is an innovation that has the potential to reach young people who may not otherwise test [3,4]. In this process, a person collects his or her own specimen (oral fluid or blood) and then performs a test and interprets the result, often in a private setting, either alone or with someone he or she trusts [5]. HIVST has been found to be an acceptable and effective strategy to increase HIV testing among marginalized populations [6,7], and the World Health Organization recommends HIVST as an approach to delivering HIV testing services to achieve the first *90* of the 90-90-90 strategy [5,8]. The Nigerian Federal Ministry of Health and the National Agency for Control of AIDS in Nigeria also recommended HIVST as a strategy to increase HIV testing uptake [9]. However, there are challenges with self-reporting of HIVST results because some people do not routinely self-report for a variety of reasons, and there is a need to enhance linkage to care following self-testing [8].

Understanding how best to reach young people in Nigeria with HIVST requires knowing whether they find HIVST feasible or acceptable. One formative study in Nigeria provided preliminary data on the willingness to engage in HIVST [10]. Moreover, in a cross-sectional survey of 157 individuals, 54.8% of participants expressed willingness to self-test, with some suggesting that HIVST will help avoid fear and stigma that may arise from the use of health facilities [11]. Similarly, in a pilot study among 257 men who have sex with men and key opinion leaders, the majority (98%) reported that it was easy to perform HIV testing with HIVST kits. Common reasons for liking HIVST were ease of use (87.3%), confidentiality and privacy (82.1%), convenience (74.1%), and absence of needle pricks (64.9%) [12]. HIVST kits became available in Nigeria in 2019. To date, only 2 HIVST kits have been introduced in Nigeria, which include the blood-based Alere HIV Combo rapid test kit and OraQuick self-test kit (saliva-based test), which is the only World Health Organization prequalified HIVST test in the country [13]. Despite the high rates of acceptability and willingness to use HIVST, we are not aware of any studies that have assessed how to improve access and reach young people with HIVST in Nigeria.

The need for this improved access for young people to HIVST kit in Nigeria prompted our youth-participatory research to increase HIVST among at-risk youth [13]. According to the Demographic and Health Survey in Nigeria 2018, 87.9% of Nigerian households own mobile phones [14]. It has also been documented that expanding access to mobile health (mHealth) technology via the use of mobile devices has increased the opportunities to deliver health services to improve health outcomes [15-17]. Building on this infrastructure, mobile apps have the potential to (1) reach the largest number of young Nigerians with HIV who remain undiagnosed, (2) create demand for HIV prevention and increase HIV test coverage, and (3) facilitate linkage to treatment for young people who test HIV-positive and provide tailored prevention strategies for those who test HIV-negative.

In response, we partnered with Co-Creation Hub, Nigeria’s first innovation laboratory and preincubation space for social tech ventures, to cocreate an HIVST mobile app targeting young Nigerians. The aim of the mobile app is to improve the verification of HIVST results and provide linkage to youth-friendly health services to young people who test for HIV. To accommodate the preferences and priorities of Nigerian youth, we engaged youth in the design and development of mobile HIVST apps. In this study, we described the development of the app and assessed the user experience. The findings will inform the implementation of mHealth HIVST services tailored to the needs of young people in Nigeria.

## Methods

### Development of the 4 Youth By Youth HIVST App

The Innovative Tools to Expand HIVST collaboration between the Nigerian Institute of Medical Research, Saint Louis University, New York University, and the University of North Carolina at Chapel Hill, known locally as 4 Youth By Youth (4YBY), engaged a team of developers at the Co-Creation Hub to develop the 4YBY photo verification mHealth app. The 4YBY photo verification mHealth app aims to promote HIVST by guiding users (youth) through the proper use of the HIVST kit and linking them to care and anonymous counseling. The mobile app was built with React Native to support the Android operating system from version 4.4 (Kitkat) upward. The development and design process were based on the Human-Centered Design framework. The design team worked together to solve 2 main challenges in creating the app: (1) how might we connect young people to health care and counseling when self-testing for HIV? (2) How can data on HIVST be collected and analyzed?

### Some of the Key Activities in the Design Process

First, interviews were held with project key stakeholders, including potential users, with the goal to understand the facilitators and constraints or major concerns with HIVST. Second, there were immersion and shadowing activities with potential users in which designers sought to understand and gain real-world experience with HIVST to understand end users’ perception and preferences of HIVST. The goal of this process is to identify and provide value to young people who will use the product. Third, user interface prototyping and content creation was done. The design team worked with researchers from the Nigerian Institute of Medical Research to create content for the different sections of the mobile app. Given that the vast majority of people perceive images faster than words, we used illustrations supported by text to communicate important concepts throughout the design process.

Following the design phase, the front end and back end of the app were developed. A front-end section of the app, consisting of a visual representation of the app and a back-end section, also known as the server side of an app, including communication between the database and the app, were developed. After the development of the first version of the app, alpha and beta testing was performed. α testing was performed to identify bugs before releasing the app to end users, whereas beta testing was performed by potential end users of the app (young people aged 14-24 years) to determine the usability of the app.

### The Key Features of the App

**Figure 1:**
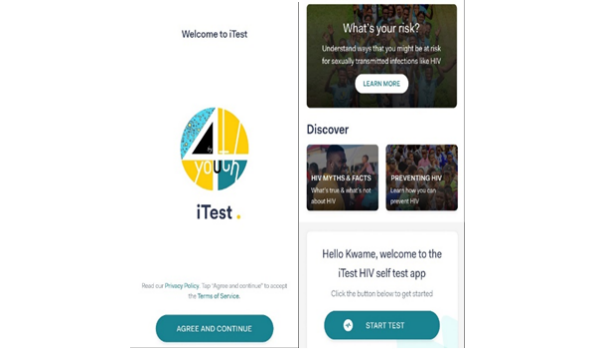
Basic information on HIV in the app.

**Figure 2:**
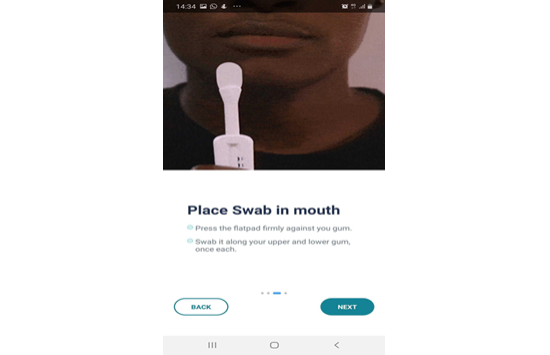
Screenshot of a video of the OraQuick swab.

**Figure 3:**
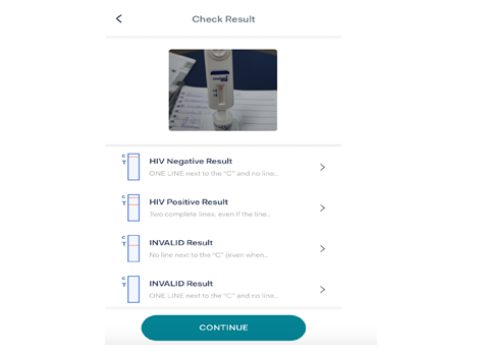
Uploaded picture of an HIV test result on the app.

The key features of the app include the following:

1. How to use the HIVST test kit: It is a combination of illustrations and written step-by-step tutorials to help users perform their tests successfully (Figures 1-3).
2. Timer and reminder: Timers and reminders are used to help end users check for test results exactly 20 minutes after sample collection.
3. Photo upload and verification: Photo upload of the test kit at the expiration of the 20 minutes for the verification of test results. The purpose of this feature is to maintain data integrity and provide photo evidence of the HIVST result performed.
4. Linkage to care: This describes the process of linking the tester to care through access to toll-free calls for postcounseling after the self-test.

Other features were as follows:

1. HIV risk assessment quiz: It is a series of questions to help assess the tester’s risk of contracting HIV and other sexually transmitted infections.
2. HIV facts and information: Educational content on HIV prevention, care, myths, and facts are included in the app that users are to review during the 20-minute period allotted for waiting for HIV test results.
3. Administrative dashboard: It is a web-based dashboard to access all data received through the mobile app. The dashboard will also allow counselors to reach out to users who have requested counseling.

### Study Design

A qualitative research design approach was used to evaluate the usability of the 4YBY mobile phone app by answering the following questions: To what extent do users achieve the desired goal of using the app (effectiveness)? Does the effort put into the use of the app allow the performance of a task in a timely manner (efficiency)? What is the perceived user’s experience of the app? Participants used a think-aloud approach while performing usability tasks in three rounds of usability testing and subsequently completed semistructured one-on-one interviews.

### Participant Recruitment

Participants were recruited through advertisements and word of mouth at university campuses and youth centers in Yaba, Lagos, Nigeria. Participants’ recruitment for the study was guided by previous mHealth app studies that showed that preliminary usability of apps can be detected by a sample of 5 to 10 individuals [18,19]. Inclusion criteria included being aged between 14 and 24 years, fluency in English, the ability to read, owning an Android electronic device, and the ability to provide informed consent for the study. Data collection was performed by researchers who were part of the study team with requisite training in good clinical practice and ethical conduct of research.

### Procedures

During each of the beta tests (Multimedia Appendix 1), participants performed HIVST using the OraQuick by Orasure testing kit. Tests were conducted by each participant swabbing their gum and then placing it in a processing fluid and waiting for 20 minutes for the result. Each participant had a prototype of the 4YBY app installed on their Android app and subsequently carried out the HIVST by following the directive provided by the app. The app also engaged the participants with basic information on HIV during the waiting time before the test completion, and they also participated in a HIV risk self-assessment test.

### Data Collection

Following informed consent, participants were involved in usability testing via the think-aloud approach [20]. The think-aloud approach required users to continuously verbalize their thoughts about their underlying thinking behind their interactions while using the mobile app by freely expressing what they were doing and why and stating when they encountered any problem in the process. This provided an opportunity for participants to test the app’s functionalities and provide opinions, comments, and concerns to improve on the app. They also discussed how they felt about the use of the app, which was audiotaped.

Immediately following usability testing, participants were asked a series of questions to assess their experience with the usability of the app (Multimedia Appendix 2). The objective of the phase was to obtain the participants’ immediate interpretation of the app design and functionality and facilitate the elaboration of usability issues and increasing insight and design suggestions [21]. A total of 3 beta tests using this methodology were performed over 3 months. After each of the beta tests, the design team used the user experience feedback to improve the development of the app by correcting all glitches noted and modifying as necessary to meet users.

### Data Analysis

The demographic characteristics of the users were reported as descriptive statistics. Each interview was audio recorded, subsequently transcribed verbatim, and manually coded. Field notes were also included in the analysis. Inductive thematic analysis, which describes the process of coding data without trying to fit the data into a pre-existing coding frame or the researcher’s analytic preconceptions, was used for this study. This process ensured that thematic analysis was completely data driven [22]. The Braun and Clark [22,23] guidelines were used by 2 research team members with experience in qualitative research, which included the following steps: (1) repeated reading of the transcripts to become familiar with the data; (2) generating initial codes relevant to the research questions (effectiveness, efficiency, and usefulness); (3) organizing the codes; (4) arranging the subthemes into overarching themes; and (5) defining and naming themes. Disagreements around themes were resolved by an in-person research team discussion until consensus was reached, and a final theme was agreed upon.

### Ethical Consideration

Ethical approval for the Innovative Tools to Expand HIVST study was obtained from the institutional review board of the Nigerian Institute of Medical Research and Saint Louis University. Informed consent was obtained from each participant before the commencement of the usability beta testing study.

## Results

### Participant Characteristics and Usability Findings

**Figure 4:**
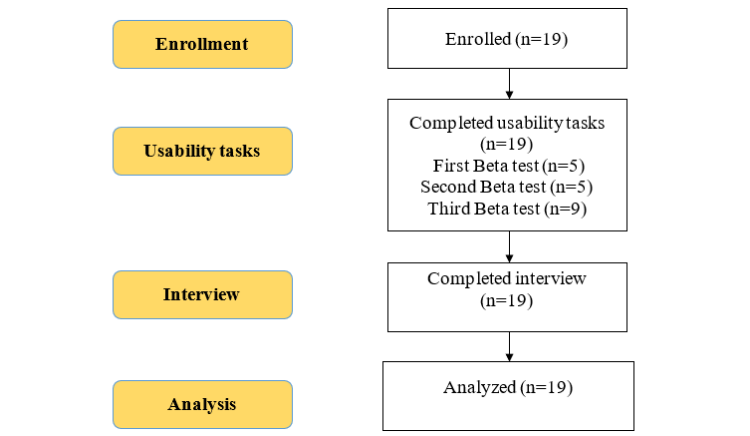
Study flow diagram.

A total of 19 youth participated in the usability analysis of the mHealth app for HIVST (Figure 4). The mean age of the participants was 22 years (SD 2.4), and the age range was 14-25 years. There were 12 females and 7 males and 68% (13/19) of the participants were students. The majority (18/19, 95%) of the participants completed all the usability tasks on the first attempt. On average, the usability test lasted between 25 and 30 minutes. All the participants successfully uploaded a photo of their test results using the app. Figure 4 illustrates participants’ progression through each of the study phases.

### User Experiences

Thematic analysis of the user comments from the think-aloud interviews from the 3 beta usability tests yielded findings on how the app was used, including the relation to test outcomes (ie, positive, negative, and invalid) and strengths and weaknesses of the prototype app and suggested improvements. The following four themes were identified (Table 1): (1) the design of the app, (2) the ease of use of the app, (3) user privacy reinforcement, and (4) linkage to care.

**Table 1:** Summary of finding from one-on-one interviews on usability of the 4 Youth By Youth app.

Variables considered	Beta test 1 (August 1, 2019)	Beta test 2 (September 24, 2019)	Beta test 3 (September 26, 2019)
Design of the app	“The App could be more attractive, with regards to color”	“The App should be more colorful/attractive” “There is a need to improve on the resolution of the videos”	“The App background should be more attractive” “There is a need to use more phrases that will catch Nigerian youth attention”
Ease of use of the app	“It is easy to use the App. The picture/videos were helpful even for a layman” “It is simple and straight forward” “Basic HIV information on the App was useful”	“The information about HIV on the App was quite educative” “The instruction on HIV self-testing on the App is adequate to safely conduct the test” “The App instruction on how to setup the test need a little more work” “I would prefer if the language of the App could also be pidgin English” “The App contain all the information I will need to do HIV self-testing on my own”	“The App is explicit, self-explanatory” “The video made it easy to understand the test process” “I wonder if the App could be used offline” “It was easy to take the photo evidence of my test” “The explanation of the test result is adequate to educate user on their test result, its significance and next steps to be taken”
App use and privacy	“It does encourage privacy”	“The App will promote privacy because many young people want to keep their health issues private” “The App is convenient especially for someone who do not want to go to health center or who live far away from health center”	“Am happy that no one can have access to my result by looking at the App on my phone”
Linkage to care after checking results	“After reading my result, I will prefer to call the health facility with the number provided”	“The feature on the App to request a call or call a youth counselor is great, though I would prefer to make the call myself after seeing my result”	“Yes I will request a call from the youth counselor if my result is positive”
Recommend the app to peers	N/A^a^	N/A	“I will recommend the App to my friends because it is simple and easy to use”
Timer countdown to result	“The time of waiting to read the result is too long.” “I will prefer an alert/buzz when the 20 minutes waiting time elapses”	N/A	“The buzz at the end of 20 minutes is important. I also like the fact that the App could function at the background while I wait for my result”

^a^N/A: not applicable.

### Design of the App

Participants frequently commented on the need for the app to have a more user-friendly design, with interactive images, an appealing color scheme, and features that are easily accessible. The participants involved in the first beta testing felt that a mobile app intended for use by youth should be colorful and attractive to ensure adequate coverage and acceptability among this age group. They further explained that the likelihood of a young person downloading the app from the Google Play store depends largely on how they perceived the app:

The App could be more attractive with regards to its color.

First round beta test participant

During the second and third beta tests, participants also wanted an improvement in the appearance of the logo of the app and its background theme. Other concerns raised during the second beta test are the need to improve the resolution of the video that describes the process of HIVST in the app:

There is a need to improve the resolution of the videos.

Second beta test participant

A participant in the third beta test also expressed a preference that the phrases used in the app should be the type that will easily catch the attention of Nigerian youths who are the end users of the app. He would prefer developers to use some locally trending phrases on social media:

There is a need to use more phrases that will catch Nigerian youth attention.

Third beta test participant

During the first beta test, some participants noted that it would be desirable to have the app work in the background while he could be engaged in the use of the mobile phone for other things. The feature was corrected before the third beta testing, and participants at the third beta testing were impressed with this feature:

The time of waiting to read the result is too long. I will prefer an alert/buzz when the 20 minutes waiting time elapses.

First beta test participant

The buzz at the end of 20 minutes is important. I also like the fact that the App could function at the background while I wait for my result.

Third beta test participant

### Ease of Use of the App

The ease of use of the app assesses the user experience of the app without direct guidance from the developers. Participants in each of the beta tests overall found the app easy to use and did not report that anything went wrong during the use of the app. Some of the other observations made on the ease of use include the need to adjust the app directive on how to set up the test and if the app could be used offline without internet access:

It is easy to use the App. The picture/videos were helpful even for a layman, It is simple and straight-forward.

First beta test participant

The instruction on HIV self-testing on the App is adequate to safely conduct the test. The app instruction on how to set up the test needs a little more work. I would prefer it if the language of the App could also be Pidgin English. The app contains all the information I will need to do HIV self-testing on my own.

Second beta test participant

The App is explicit, self-explanatory. The video made it easy to understand the test process. I wonder if the App could be used offline. It was easy to take the photo evidence of my test. The explanation of the test result is adequate to educate users on their test result, its significance, and the next steps to be taken.

Third beta test participant

### User Privacy Reinforcement

HIV stigma is a key pertinent issue with HIV testing. To ensure that all users can test anonymously, aliases, rather than real names, were used. The developer also implemented an auto-logout feature for further security. However, in think-aloud exercises, one of the major concerns shared among participants was the issue of privacy. The participants would not want their personal information to be shared in any form, and the app ensured anonymity to address this. The participants during the 3 phases of the beta test noted that the app further reinforced the practice of privacy in getting tested for HIV:

The App will promote privacy because many young people want to keep their health issues private. The App is convenient especially for someone who does not want to go to the health center or who lives far away from the health center.

Second beta test participant

Am happy that no one can have access to my result by looking at the App on my phone.

Third beta test participant

### Linkage to Services

One of the features of the 4YBY photo verification app is that the participants can be linked to care after conducting an HIV test. This is usually done by the user calling a counselor to discuss the HIV test result or requesting a call from a counselor by clicking on a radio button in the app. The users were pleased that this feature was included in the app, and they would either call or request a call to be linked to care after performing the HIV self-test:

After reading my result, I will prefer to call the health facility with the number provided.

First beta test participant

The feature on the App to request a call or call a youth counselor is great, though I would prefer to make the call myself after seeing my result

Second beta test participant

Yes I will request a call from the youth counselor if my result is positive.

Third beta test participant

## Discussion

### Principal Findings

We developed a photo verification mHealth app to promote HIVST among young people in Nigeria. To our knowledge, this is one of the first mHealth apps that has been developed to promote HIVST and linkage to care in sub-Saharan Africa [24]. Our usability findings add evidence to the limited data on young people’s experiences and perspectives on mHealth apps in sub-Saharan Africa. We identified 4 main themes (eg, app design, ease of use, user privacy reinforcement, and linkage to care) that may influence the adoption and continued use of the apps for HIVST.

This study corroborates the findings of limited studies in sub-Saharan Africa, including how mHealth apps for HIVST may overcome the experience of stigma and structural barriers of clinic-based testing by providing privacy and convenience [25]. Even in middle- to high-income countries, with a substantial body of research on mHealth apps and HIVST, findings suggest that the design of these apps, along with their ease of use, privacy, and linkage to services, provides an opportunity to disseminate information about the accuracy, safety, and appropriate use of this technology to promote HIVST [26].

Our iterative cocreation process led to the development of an mHealth HIVST app suited to the needs of young people in Nigeria. In addition, the iterative process and feedback from beta testing led to the modification of the app to address issues raised during the development phase, including the desire to have an app with minimal internet data demand, features to encourage users’ anonymity, and step-by-step video description of the process of conducting the HIVST. Previous studies on mHealth app development have documented the importance of involving end users in the development of mHealth apps [27]. Through their own experiences and by showing participants how to use our app, the majority found the app to be informative, accessible, and engaging. Think-aloud activities identified not only facilitating features that may potentially enhance uptake but also factors that may hinder its use.

Consistent with other mHealth app literature [28,29], one primary recommendation was for the app to reinforce privacy concerns. This is particularly salient for young people, given their limited access to youth-friendly health centers and barriers associated with navigating and appearing at clinics for HIV prevention services that mostly cater to adults [30,31]. Protecting the privacy of young people accessing the app will not only maintain youth support but also enhance trust. Providing linkage to additional services via the app also provides an opportunity to further enhance youth engagement with HIV prevention, treatment, and care.

Participants noted that the app’s linkage platform can enable HIV services to reach young people at risk for or living with HIV, who may not otherwise use health care services. In addition, to the linkage to services, participants reported that the use of multimedia text, short videos, and graphics within the app may not only help youth to remain HIV-uninfected but also enhance their ability to stay healthy and thrive if they are already living with HIV. Overall, these findings provide insight into young people’s concerns and strategies to enhance the uptake of our mHealth app for HIVST promotion.

This study had some limitations. First, this was a small sample of youth participants recruited from Lagos, Nigeria, thereby making it difficult to reach generalizations from this data source. Second, access to smartphones and mHealth apps may have influenced social desirability bias with the decision to participate in the study. In addition, the flexibility of thematic analysis can lead to inconsistency and a lack of coherence with theme development. To address authors’ bias, the coding of the transcript was independently performed by 2 researchers before theme development. Finally, given that this is a usability study, it is not possible to conclude the potential efficacy of the app in promoting HIVST uptake among young people in Nigeria.

The results of this study have implications for interventions targeting HIVST uptake among young people in Nigeria. It also has important implications for using technology to disseminate HIV prevention information to youth populations in Nigeria. The 2019 Nigerian operational guidelines for HIVST seek to increase individuals’ autonomy, decentralize services, and create demand for HIV testing, especially for underserved populations, including young people. Given the new operational guidelines that seek to decentralize HIV testing, verification of self-reported outcomes for HIVST is of utmost importance. Bearing in mind that there are no available photo verification modalities for HIVST for young people in Nigeria, this study provides evidence on the usability of the 4YBY mHealth photo verification app and its potential to promote uptake of HIV testing among young people. These findings lay the foundation for future studies to assess the feasibility, reach, and effectiveness of the mHealth app to promote the uptake of HIV testing and other preventive services among young people in Nigeria. Through this formative work, we also obtained contextual recommendations from the participants to improve the design of the app and reinforce privacy to enhance acceptability among young people in Nigeria. The feedback has practical implications for mHealth technologies to seek end users’ feedback to ensure acceptability and appropriateness among target populations. The 4YBY mHealth app may provide a model for supporting young people to access other sexual and reproductive health services, given the app’s built-in function to link users to available youth-friendly health facilities. The findings have implications for practice in Nigeria and can inform the integration of mHealth technologies within youth-friendly health facilities to promote the uptake of preventive sexual health services.

### Conclusions

This study demonstrated the usability of an mHealth app to promote HIVST uptake among young people in Nigeria. Findings from this study illustrated that the design and ease of use of the app, along with privacy and linkage to additional services, may enhance the reach and acceptability of HIVST services among young people who are traditionally underserved in traditional HIV research conducted not only in Nigeria but also in sub-Saharan Africa. Future clinical trials are needed to determine the efficacy of using an mHealth app to verify HIVST results among young people.

## Appendix

Multimedia Appendix 1 App process flow (usability task flowchart).

Multimedia Appendix 2 Usability questionnaire.

## Abbreviations

4YBY: 4 Youth By Youth
HIVST: HIV self-testing
mHealth: mobile health
