# Supplementary figures and images for: The 4 Youth By Youth mHealth Photo Verification App for HIV Self-testing in Nigeria: Qualitative Analysis of User Experiences
Source: JMIR Form Res. 2021 Nov 17;5(11):e25824. doi: 10.2196/25824 (PMC8663582; doi:10.2196/25824)

Multimedia Appendix 1. Application process flow (Usability Task Flowchart)

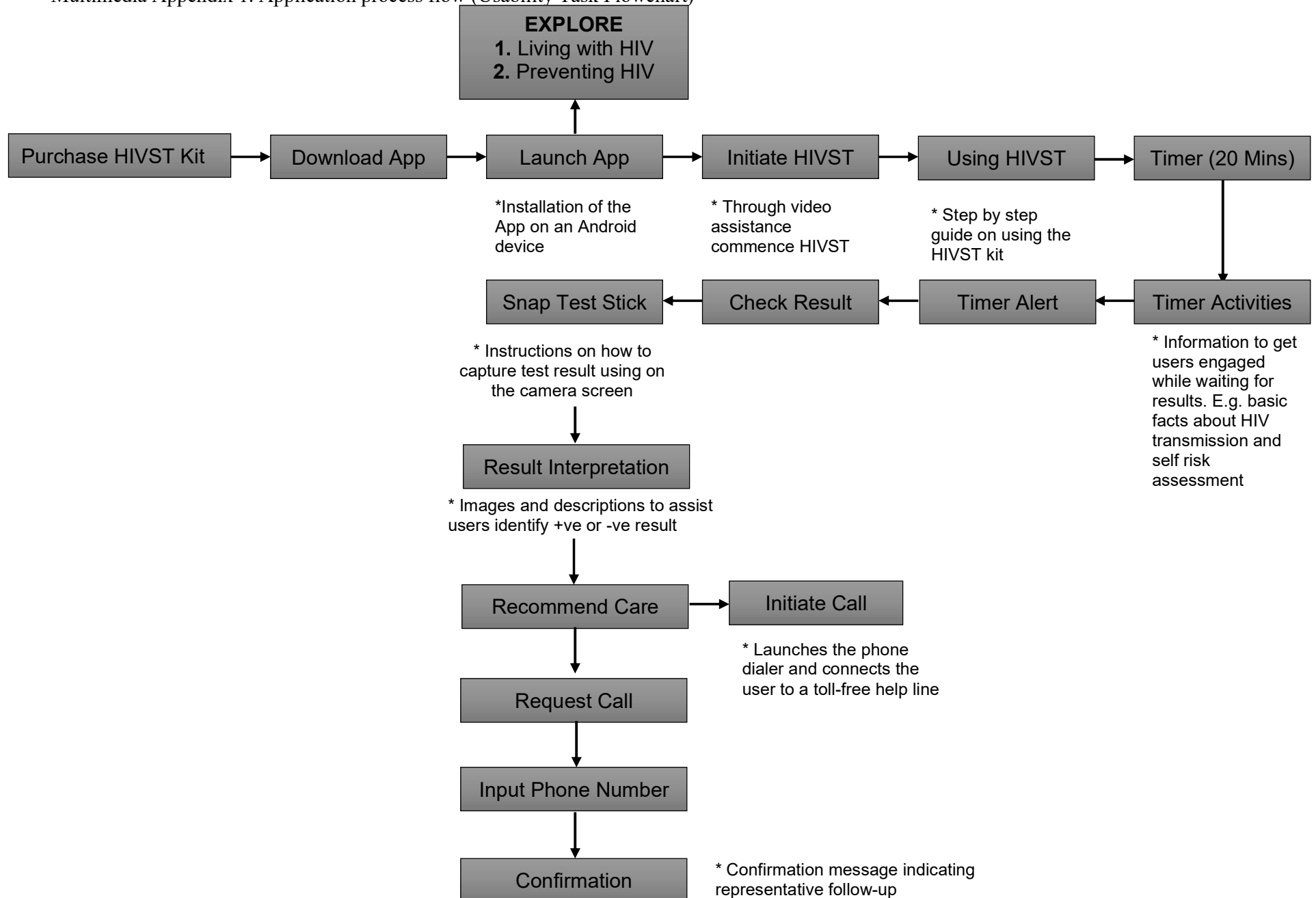

Supplement: Multimedia Appendix 1 [file formative_v5i11e25824_app1.pdf]
