# Supplementary material for: The 4 Youth By Youth mHealth Photo Verification App for HIV Self-testing in Nigeria: Qualitative Analysis of User Experiences
Source: JMIR Form Res. 2021 Nov 17;5(11):e25824. doi: 10.2196/25824 (PMC8663582; doi:10.2196/25824)
Supplement: Multimedia Appendix 2 [file formative_v5i11e25824_app2.docx]

**Usability Questionnaire**

Thank you for accepting to participate in the usability study of the 4yby Photo verification mobile app for promoting HIV self-testing among adolescents in Lagos, Nigeria.

A] Screening/Introduction

1. How old are you(age last birthday)…………………..
2. What is your level of education Primary [ ]; Secondary [ ], Tertiary [ ]
3. What is your gender? Male [ ]; Female [ ]

B] You have used the 4YBY Photo-verification app for HIV self-testing. Can you briefly answer the following question about your experience using the app.

1. Tell us your overall experience of the mobile app
2. Specifically, what do you like or dislike about the outlook and your design of the app
3. What is your opinion about whether the app was easy to use or not?
4. One of the reasons for developing the app is to promote HIV self-testing which is the process of collecting personal sample, testing for HIV personally and interpreting the result. What is your opinion about privacy when you used the app?
5. When tested for HIV using the app, do you think the app provide you with the option of asking further questions of getting further care
6. What is your overall perception of the app.
7. If you were to change anything about the app, what will it be?
